# Combined bioinformatics technology to explore pivot genes and related clinical prognosis in the development of gastric cancer

**DOI:** 10.1038/s41598-021-94291-5

**Published:** 2021-07-29

**Authors:** Jiasheng Xu, Xinlu Wang, Qiwen Ke, Kaili Liao, Yanhua Wan, Kaihua Zhang, Guanyu Zhang, Xiaozhong Wang

**Affiliations:** 1 Department of Clinical Laboratory, The Second Affiliated Hospital of Nanchang University, No. 1 Minde Road, Nanchang, 330006 Jiangxi China; 2 Public Health College of Nanchang University, Nanchang, China; 3 Information Engineering School of Nanchang University, Nanchang, China; 4 Department of General Surgery, The Jiujiang Affiliated Hospital of Nanchang University, Jiujiang, China; 5 Jiangxi Province Key Laboratory of Laboratory Medicine, Department of Clinical Laboratory, The Second Affiliated Hospital of Nanchang University, No. 1 Minde Road, Nanchang, 330006 Jiangxi China

**Keywords:** Molecular biology, Oncology

## Abstract

To screen the key genes in the development of gastric cancer and their influence on prognosis. The GEO database was used to screen gastric cancer-related gene chips as a training set, and the R packages limma tool was used to analyze the differential genes expressed in gastric cancer tissues compared to normal tissues, and then the selected genes were verified in the validation set. The String database was used to calculate their Protein–protein interaction (PPI) network, using Cytoscape software's Centiscape and other plug-ins to analyze key genes in the PPI network. The DAVID database was used to enrich and annotate gene functions of differential genes and PPI key module genes, and further explore correlation between expression level and clinical stage and prognosis. Based on clinical data and patient samples, differential expression of key node genes was verified by immunohistochemistry. The 63 characteristic differential genes screened had good discrimination between gastric cancer and normal tissues, and are mainly involved in regulating extracellular matrix receptor interactions and the PI3k-AKT signaling pathway. Key nodes in the PPI network regulate tumor proliferation and metastasis. Analysis of the expression levels of key node genes found that relative to normal tissues, the expression of ITGB1 and COL1A2 was significantly increased in gastric cancer tissues, and patients with late clinical stages of tumors had higher expression of ITGB1 and COL1A2 in tumor tissues, and their survival time was longer (*P* < 0.05). This study found that ITGB1 and COL1A2 are key genes in the development of gastric cancer and can be used as prognostic markers and potential new targets for gastric cancer.

## Introduction

Gastric cancer is one of the common malignant tumors of the digestive system, and its global morbidity and mortality rates are high^1^. Most patients with gastric cancer are already in the middle and advanced stages of the disease at the time of consultation, and are prone to recurrence and metastasis after surgery. Chemotherapy can help to prolong the survival time of patients and improve the quality of life of patients. However, the efficacy of chemotherapy for gastric cancer is currently limited. Finding new therapeutic targets and developing effective chemotherapy drugs are hot issues in the treatment of gastric cancer^2,3^.

Some studies have shown several differentially expressed genes in the development of gastric cancer, which might be the potential targets of anti-cancer therapy or diagnostic markers in the future^4,5^. The paradigm of network is of great importance for systems biology. It represents a powerful tool for describing and analyzing complex systems, their elements and connections, with the ability to examine the underlying topologies, structures, architectures, and functions emerging from the arrangement of components^6^. In recent years, network medicine has gained increasing attention from the scientific community. It holds that a disease is caused by disturbances of the interactome or all the physical interactions within a cell^7^. Network medicine relies on different kinds of networks: from the molecular level of protein–protein interactions (PPI) to gene regulatory network and correlation studies of gene expression^8^. In a word, network medicine provides a platform, which helps to systematically explore not only the molecular complexity of a particular disease, leading to the identification of disease modules and pathways, but also the molecular relationships between distinct pathological phenotypes^9^. The exploitation of network medicine enables many potential biological and clinical applications. It is of great significance to explore the key genes in the development of gastric cancer and their influence on prognosis with the help of network.

Based on these, we used the GEO database (https://www.ncbi.nlm.nih.gov/geo/) Screen gastric cancer gene chip, and used bioinformatics technology to establish differential gene expression profiles of gastric cancer. Combining with clinical data, we can analyze the biological function of differential genes and their impact on clinical prognosis. Through finding key genes in the development of gastric cancer and their potential molecular mechanisms that affect prognosis, we can provide theoretical basis for finding new targets for gastric cancer treatment.

## Experimental method

### Gene chip screening

The GEO database (https://www.ncbi.nlm.nih.gov/geo/) was used to screen human gene chips containing gastric cancer tissues and normal tissues, with the numbers GSE26899 (T = 96, N = 12), GSE26942 (T = 205, N = 12), GSE33651 (T = 40, N = 12), GSE54129 (T = 111, N = 21). Download the matrix file of the above gene chips to obtain sample information.

### Screening and processing of differential genes (DEGs)

Using R packages limma, the differential genes between gastric cancer tissues and normal tissues were screened in the above four gene chips, and gastric cancer was screened out according to the conditions of adj.P. Val < 0.05, 2^|log2 (FC)| > 1. Differential genes were significantly up-regulated and down-regulated in tissues compared to normal tissues, and a Wayne diagram was performed on the differential genes of the GSE26899, GSE26942, and GSE33651 gene chips, and the gene chip GSE54129 was used as a validation set to examine the expression value and cluster discrimination of the differential genes screened. Using GSE54129 sample type and gene expression, R package ggplot2 was used to draw the volcano map, and R packages heatmap was used to draw the gene cluster heatmap.

### Protein–protein interaction (PPI) network construction and analysis

Input all genes into the STRING database (https://string-db.org/) to obtain the PPI network of gene interactions. The confidence score was set as 0.9, and the confidence score was used as the standard for protein inclusion in the network (so that only interactions that higher than this score could be included in the protein network) to obtain PPI network and calculate nodes. Use Cytoscape3.6.0 (http://www.cytoscape.org/) software to screen Hub genes: Click "Apps" at the top of the main screen, click "Apps Maneger" to download and install the "Cytohubba" package. To judge the degree of a node in the whole network by the number of connections between a node and other nodes, and to rank the nodes in the network by the network function. Select the content of "Target Network", select the MCC mode, and calculate the "Nodes Scores" of each protein node. Use the mean of each protein node’s degree value as the threshold value of the core node of the PPI network. Screen out proteins whose degree value was greater than the threshold (the mean value of degree value of all proteins), these proteins served as the key nodes of PPI network^10,11^ and calculate the correlation score between the nodes and their interacting proteins.

### Differential gene function enrichment and annotation

The gastric cancer differential genes were introduced into the DAVID database (https://david.ncifcrf.gov/). The biological functions of the differential genes were annotated according to the GO (gene ontology) database^12^, and the selected gastric cancer differential genes were introduced into KEGG (Kyoto Encyclopedia of Genes and Genomes) database for KEGG pathway enrichment analysis, and Cytoscape plugin ClueGO was used for enrichment analysis of target genes’ functions and pathways.

### Survival analysis of key nodes

In order to further verify the key role of ITGB1 and COL1A2 proteins in the PPI network in the malignant progression of gastric cancer, the GEPIA online website was used to analyze the differences in the expression levels of pivot genes in gastric cancer samples and normal tissues, and to further analyze the pivot genes in gastric cancer patients.

Correlation analysis of the selected pivot genes was performed using the GEPIA online website. Pearson was used as the correlation coefficient to analyze the correlation of the pivot genes in gastric cancer tissues and normal tissues. Using the GEPIA analysis tool to analyze the prognosis of target genes from the data of gastric cancer samples in the Tumor Genome Atlas (TGGA) database, and explore the impact of ITGB1 and COL1A2 on the survival and prognosis of gastric cancer.

### Gastric cancer tissue chip and immunohistochemical staining

20 patients diagnosed with gastric cancer undergoing parallel tumor resection were screened from our hospital. Patients were all screened for gastric adenocarcinoma with pathological grade II and clinical stage II or III. All patients were confirmed by pathological examination and diagnosed by two senior pathologists. Tissue chips were taken from their tumor tissues and corresponding adjacent tissues to collect tissues. Tissue chip was dewaxed for 20 min in xylene, and then in fresh xylene. Repeat 1 time. Soak the dewaxed chip in 100% ethanol for 5 min twice, and soak each of 95% ethanol, 80% ethanol, and distilled water for 5 min. Basic antigen repair solution (Tris-E DTA, pH 9) was used. Use a pressure cooker to boil, place the chip, time it for 2 min, and naturally cool to room temperature. Incubate for 10 min at room temperature in the dark with 3% H2O2, block with normal sheep serum working solution for 30 min at room temperature. Then add primary antibody RP215, 4 °C overnight, HRP-labeled secondary antibody at room temperature for 30 min. DAB coloration, hematoxylin counterstaining. Microscopic observation, randomly selected 5 high-power fields, and two pathologists independently read the film. Cytoplasmic staining score uses four intensity levels (0: negative, 1: weak positive, 2: moderate positive, 3: strong positive) and percentage of positive cells (0: 0%, 1: 1%–5%, 2: 6%–25%, 3: 26%–50%, 4: 51%–100%). The final score is product of intensity grade and positive cell rate (percentage) grade, 0–3 for low expression and 4–9 for high expression.

### Statistical analysis

Data analysis was performed using SPSS 20.0 statistical software. Comparison of cancer-Ig G expression levels in different tissues was performed using the χ^2^ test and independent sample t test, and clinical pathological characteristics were analyzed using the χ^2^ test. Survival analysis was performed using Kaplan–Meier method. P < 0.05 was defined as a statistically significant difference.

### Ethical approval and consent to participate

This study was carried out in accordance with the recommendations of the Ethics Committee of the Second Affiliated Hospital of Nanchang University. The protocol was approved by the Ethics Committee of the Second Affiliated Hospital of Nanchang University. All subjects gave written informed consent in accordance with the Declaration of Helsinki.

## Results

### Gene chip sample information

Download the matrix files of gastric cancer-related gene chips (numbered GSE26899, GSE26942, GSE33651, GSE54129) from the GEO database. The statistical results of the sample clinical information were shown in Table 1.

**Table 1:** Basic clinical data of patients included in the study.

Clinic pathologic variables	n
**Age**	
≤ 40	23
> 40, ≤ 60	173
> 60	145
Unknown	168
**Gender**	
Male	243
Female	98
Unknown	168
**Tissue types**	
Tumor	452
Normal	57
**Lauren classification**	
Diffuse	73
Intestinal	200
Mixed	9
Unknown	227
**Ajcc stage**	
1	60
2	56
3	90
4	87
Unknown	216
**Adjuvant chemotherapy**	
Yes	178
No	95
Unknown	236

### Analysis and screening of differential genes

**Figure 1:**
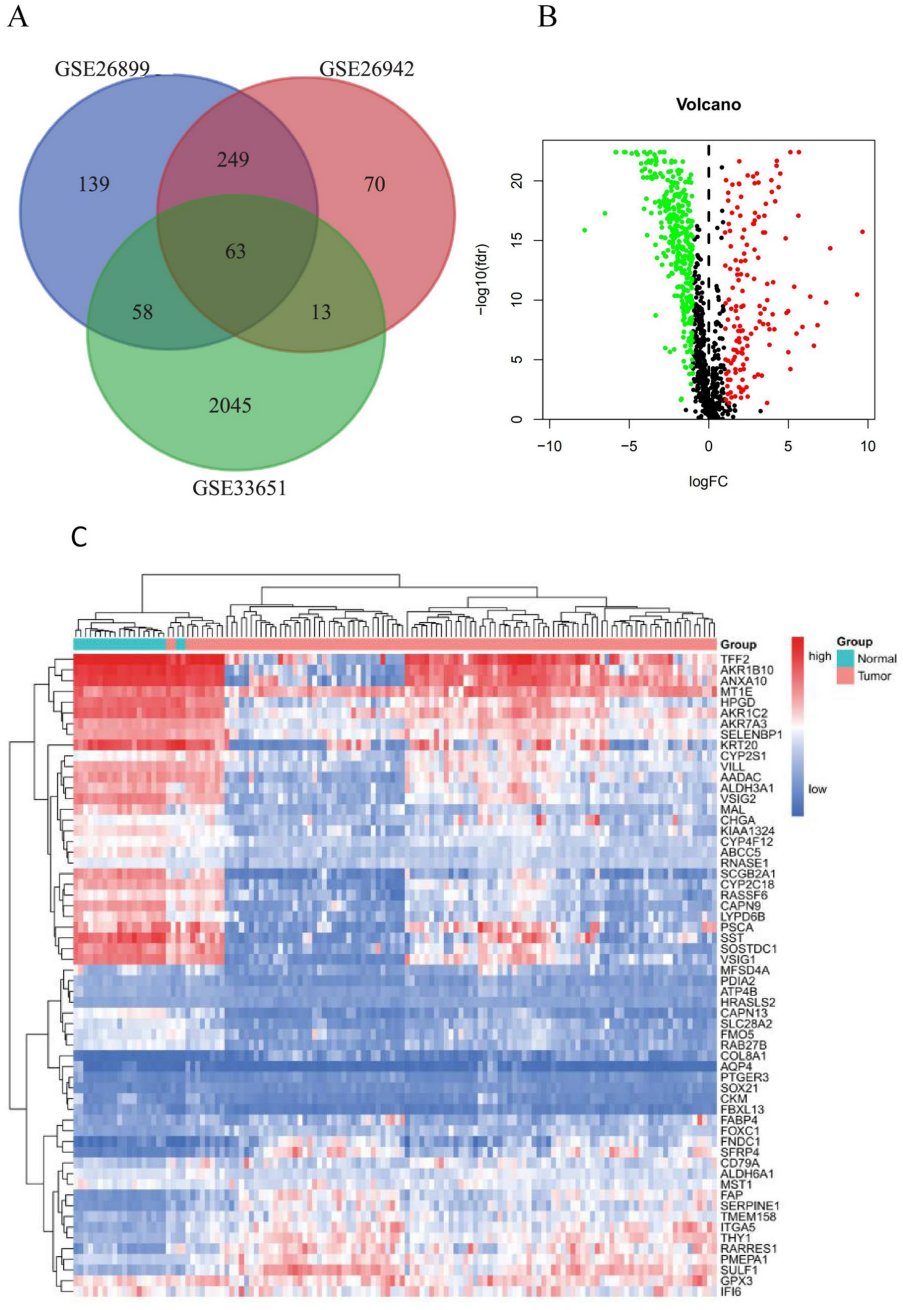
(**A**) The Venn plot of differentially expressed genes (drawn by VennDiagram R package 1.6.20, https://CRAN.R-project.org/package=VennDiagram); (**B**) Volcano map of differentially expressed genes (drawn by ggplot2 R package 3.3.2, https://ggplot2.tidyverse.org/); (**C**) Clustering heatmap of differentially expressed genes (drawn by heatmap R package 1.1.9, https://CRAN.R-project.org/package=heatmap3).

Differential gene analysis showed that a total of 63 genes had significant changes in gastric cancer tissues of the three groups of chips (Fig. 1A). The above genes were then used to cluster the validation set GSE54129 gene chip, and the volcano map and heat map results showed that the screened differential genes had good discrimination between normal tissues and gastric cancer tissues (Fig. 1B,C). Fig. 1A: The Venn plot of DEGs; Fig. 1B: Volcano map of DEGs; Fig. 1C: Clustering heatmap of DEGs.

### Construction and analysis of PPI network

**Figure 2:**
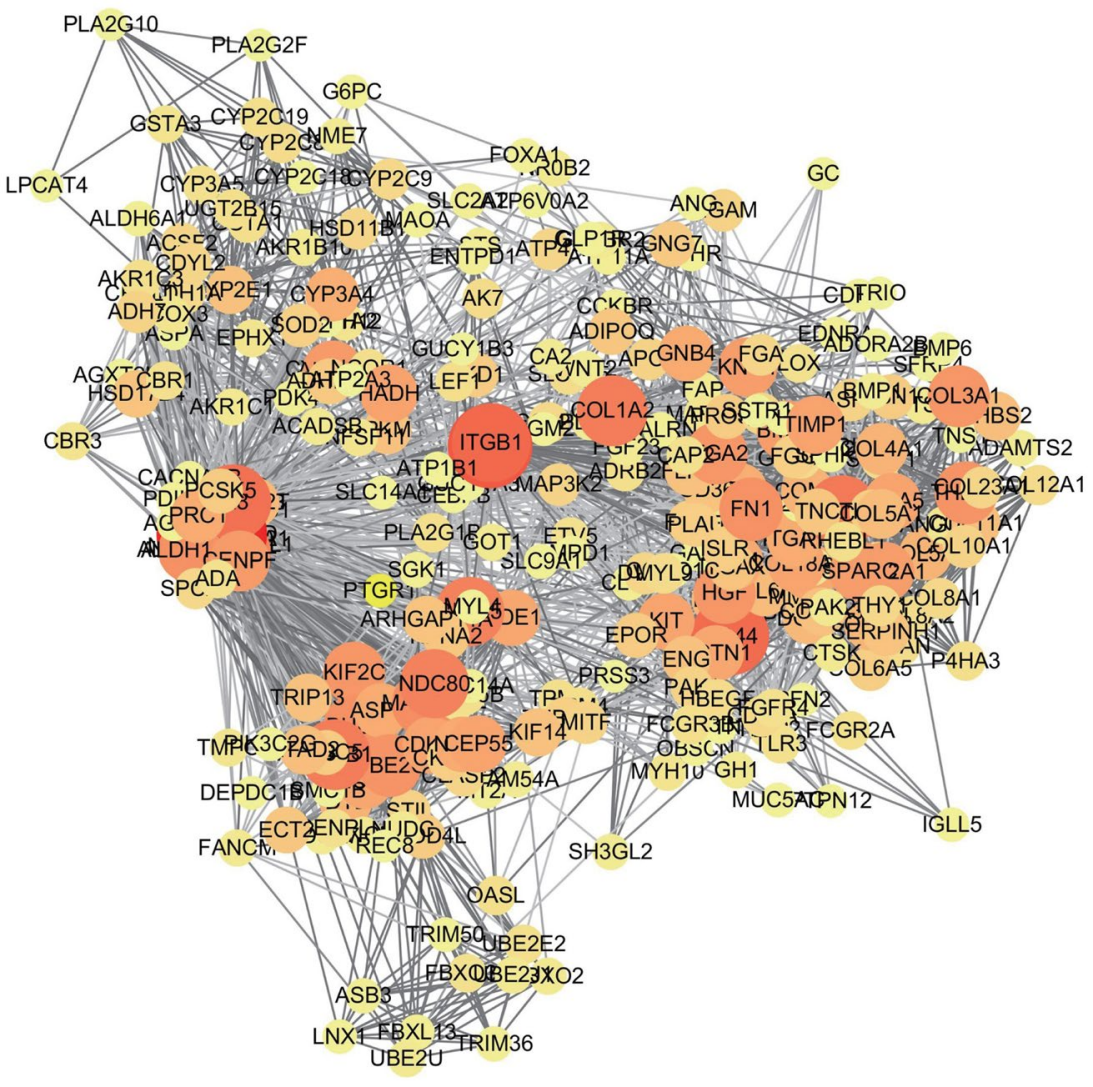
PPI Network plot of differentially expressed genes (drawn by the STRING database 11.0, https://string-db.org/).

Use the String database (https://string-db.org/) to construct a PPI network of characteristic genes, and calculate the nodes. As shown in Fig. 2, the greater the Degree value of a node in the figure, the darker its color and the larger its diameter. Define the nodes whose degree were greater than the threshold value of 9.686 as the ITGB1 and COL1A2 were the key nodes of this module. Further analysis of the interaction between the nodes of the PPI network revealed that 31 proteins in the characteristic genes of gastric cancer were connected to the ITGB1 protein. Among them, 19 had scores greater than 0.9, and the top 5 were: ITGA5, ITGA1, CD9, ITGAX, ITGA2; the interaction analysis of another key node COL1A2 found that there were 27 proteins connected to COL1A2, of which 11 had scores greater than 0.9. The top 5 were COL1A1, COL6A3, LUM, ADAMTS2, ITGA2.

### Functional enrichment and pathway enrichment of differential genes in gastric cancer

**Figure 3:**
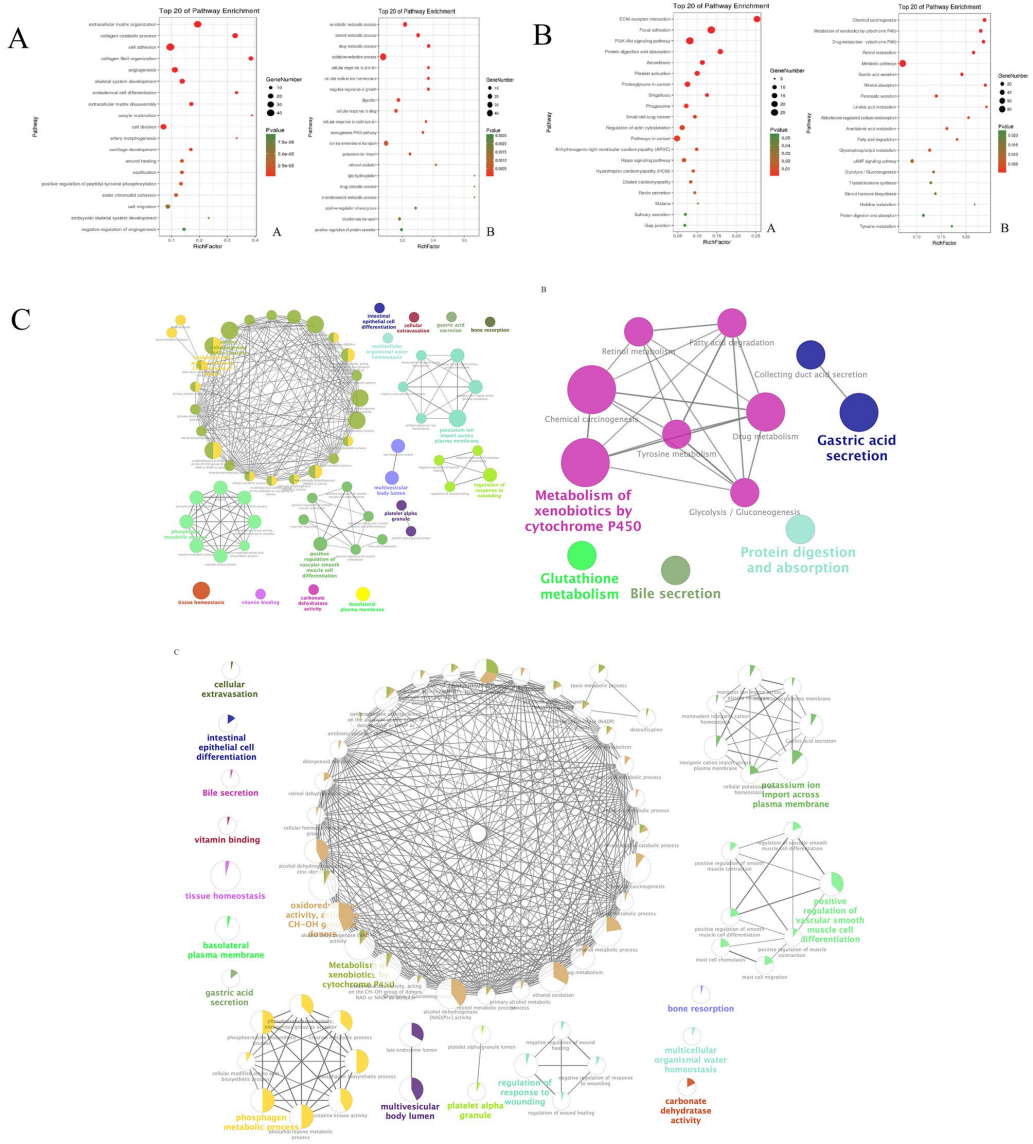
(**A**) The top 20 GO enriched by DEGs (drawn by ggplot2 R package 3.3.2, https://ggplot2.tidyverse.org/); (**B**) The top 20 KEGG pathways enriched by DEGs (drawn by ggplot2 R package 3.3.2, https://ggplot2.tidyverse.org/); (**C**) ClueGO network of pathways for DEGs (drawn by Cytoscape 3.6.0, http://www.cytoscape.org/).

The DAVID database was used to enrich and annotate the differential genes separately. The results of GO biological function annotation were shown in Fig. 3A: Genes up-regulated by gastric cancer compared with normal tissues were mainly involved in regulating extracellular matrix, collagen metabolism, cell adhesion, and collagen fibrils, tissues, angiogenesis and other biological processes; Down-regulated genes in gastric cancer were mainly involved in regulating biological processes such as redox processes and exogenous metabolic processes. The enrichment results of the screened differential genes for gastric cancer were shown in Fig. 3B. The up-regulated genes were mainly enriched in extracellular matrix receptor interactions, focal adhesions, PI3K-AKT signaling pathway, protein digestion and absorption and other signaling pathways. Down-regulated genes were mainly enriched in signal pathways such as chemical carcinogenesis, exogenous substance metabolism of cytochrome P450, and retinol metabolism. The ClueGO method was further used to analyze the biological functions involved in key factors, and it was found that the differential genes of gastric cancer were concentrated in nuclear chromosome separation, platelet degranulation, cell adhesion, and regulation of cMAP biosynthesis (Fig. 3C).

### Expression of key nodes in gastric cancer and their correlation with prognosis

**Figure 4:**
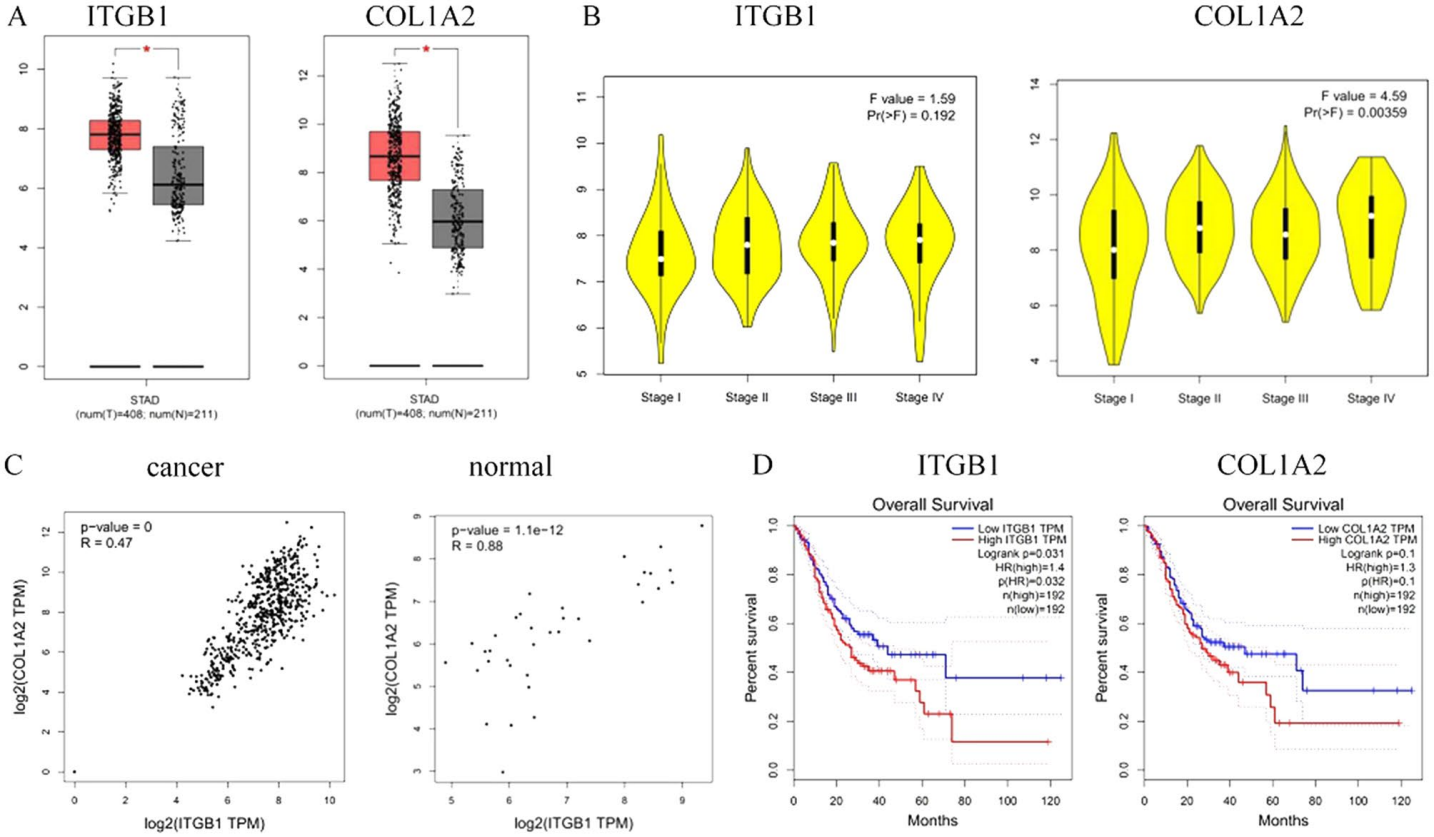
(**A**) Expression of pivot genes in gastric cancer tissues and normal tissues (drawn by GEPIA 1.0, http://gepia.cancer-pku.cn/index.html); (**B**) Expression of each hub gene in different clinical stages of gastric cancer patients (drawn by GEPIA 1.0, http://gepia.cancer-pku.cn/index.html); (**C**) Correlation between the expression of two target genes in gastric cancer tissues and adjacent normal tissues (drawn by GEPIA 1.0, http://gepia.cancer-pku.cn/index.html); (**D**) Relationship between target gene expression and survival of patients with gastric adenocarcinoma (drawn by GEPIA 1.0, http://gepia.cancer-pku.cn/index.html).

The GEPIA online website (Gene Expression Profiling Interactive Analysis) analysis found that the expression levels of pivot genes in gastric cancer samples were much higher than normal tissues (Fig. 4A). They also further analyzed the expression of each pivot gene in different clinical stages of gastric cancer patients. It was found that the patients whose tumors were clinically staged later had higher expression levels of ITGB1 and COL1A2 (P < 0.05) (Fig. 4B). Analysis of the correlation between the expression of the two target genes in normal tissues of gastric cancer patients revealed that the graph was scatterring distributed, there was no obvious correlation. In gastric cancer tissues, the expression of the two is positively correlated, and all points are highly aggregated and linearly distributed (Fig. 4C). It can be speculated that two genes may be involved in the occurrence and development of gastric cancer at the same time. And the expression showed a positive correlation trend. GEPIA was used to analyze the survival correlation of the nodes with higher degree values in the PPI network, and to examine the relationship between the expression level and the survival rate of patients with gastric adenocarcinoma.

The ITGB1 node degree value was 67, and patients with gastric cancer expressing more ITGB1 had significantly lower survival rates than those with low ITGB1 expression, *P* = 0.022. Similarly, the COL1A2 node Degree value was 57. Gastric cancer patients with high expression of COL1A2 had significantly lower survival rates than patients with COL1A2 low expression, *P* = 0.029 (Fig. 4D).

### Experimental verification of key node expressions in gastric cancer

**Figure 5:**
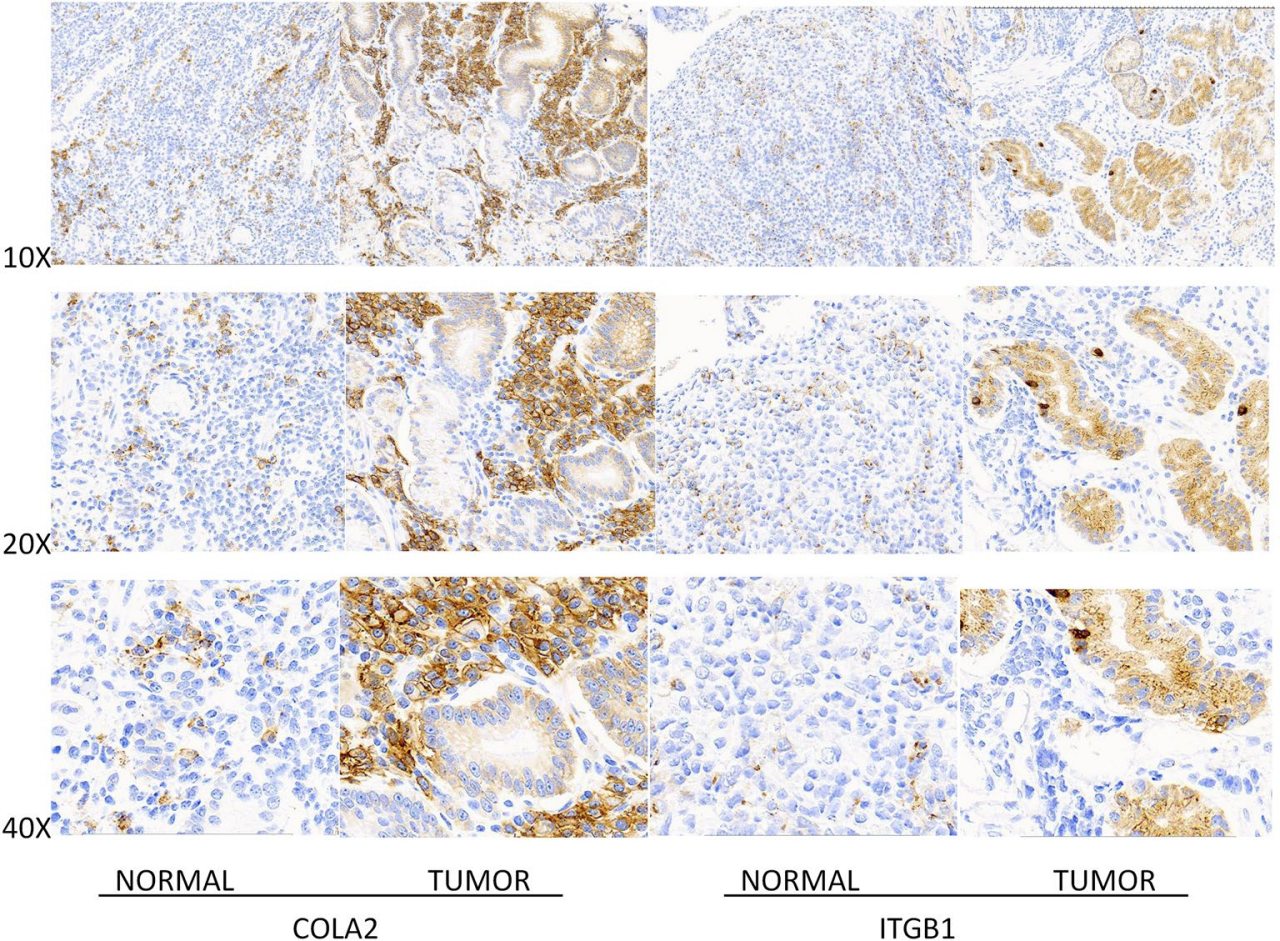
Immunohistochemical detection results of the expression of two target genes in gastric cancer tissues and adjacent normal tissues. The results suggested that compared with the normal tissue, the expression of ITGB1 and COL1A2 in gastric cancer tissues were significantly increased.

There were 12 men and 8 women with a mean age of 57 ± 1.8 years in the patients. Through immunohistochemical analysis of tumor samples and adjacent tissues of gastric cancer patients, the results showed that in normal tissues adjacent to cancer, the average score of ITGB1 expression was 1.6 points, and the average score of COL1A2 expression was 2.2 points. In gastric cancer tissues, the average score of expression of ITGB1 was 7.8, and the average score of expression of COL1A2 was 7.0. Compared with normal tissues adjacent to cancer, the expression of ITGB1 and COL1A2 in gastric cancer tissues were significantly increased (Fig. 5).

## Discussion

Gastric cancer is one of the most common malignant tumors, and its occurrence and development is a complex biological process involving multiple factors. At present, the pathogenesis of gastric cancer is not clear, and there are many challenges in treatment. The FOLFOX regimen (fluorouracil, oxaliplatin, Calcium folic acid) is currently the first-line chemotherapy regimen for gastric cancer, but chemotherapy resistance and peritoneal metastasis often appear in the clinic application^13,14^. In recent years, the development of gastric cancer-targeted drugs and treatment strategies has made certain progress. Studies have found that gastric cancer cells showed periodic mutations in some key oncogenes such as Her2, epidermal growth factor receptor (EGFR), PI3K, mTOR, or c-Met^15^. However, both traditional chemotherapy and targeted therapy drugs will cause drug resistance during the treatment process. Researches on chemotherapy resistance is still in the bottleneck stage, and no breakthrough has been achieved^16,17^. Therefore, the study of gastric cancer malignant mechanism has important scientific significance. This study obtained gene chip analysis of 509 cases of normal tissues and gastric cancer through GEO database search. Through analysis of differential expression profiles of gene chips of normal tissues and gastric cancer tissues, the key differential genes of gastric cancer were screened out. Then, we achieved construction of PPI network, calculation the key nodes of PPI and analysis the protein–protein interaction relationship. The relationship between PPI nodes and gastric cancer survival rate was further investigated. Based on these, functional annotation and pathway enrichment of differential genes in gastric cancer were performed.

This experiment first verified the abnormal expression of differential genes in gastric cancer tissues, then constructed a PPI network, calculated its key nodes as ITGB1 and COL1A2, and finally examined its correlation with the survival rate of patients with gastric adenocarcinoma. The study found that high ITGB1- and COL1A2-expression patients with gastric cancer had significantly lower survival rates than those with low expression of ITGB1 and COL1A2, which indicates that ITGB1 and COL1A2 are closely related to the occurrence and development of gastric cancer and their malignant progression. ITGB1 is a member of the integrin family and is involved in regulating cells adhesion, metastasis and growth of tumor cells. ITGB1 is involved in regulating the invasion, angiogenesis and metastasis of various epithelial malignancies such as gastric cancer, breast cancer and glioblastoma^18,19^. It is reported that ITGB1 is more sensitive to mark the early gastric cancer^20^. Based on the PPI network, this study found that cd9 may have a potential regulatory effect on ITGB1. Although cd9 has been studied to regulate the malignant progress of gastric cancer, its relationship with ITGB1 has not been reported in the literature. COL1A2 is type I collagen Protein α-2 (I) chain, type I collagen is the main component of extracellular matrix (ecm), and some studies have found that compared with precancerous lesions, COL1A2 in gastric cancer group may be significantly up-regulated, and it may be used as a marker for gastric cancer prediction and early cancer determination^21^.

This group analyzed protein interactions through PPI network and found that proteins such as lum and itga2 had strong interactions with COL1A2, although there were few studies believing that it can participate in the regulation of fibroblast extracellular matrix synthesis, and its relationship with the COL1A2 gene has not been reported in the literature. Moreover, Adamts2 has not been studied to explore its interaction with COL1A2.

Further enrichment analysis of gastric cancer differential genes found that gastric cancer tissues had significant differences in molecular characteristics and biological behavior, especially some genes related to the tumor microenvironment, from normal tissues. On the one hand, the gastric cancer differential genes are functionally concentrated in the extracellular matrix, collagen metabolism, cell adhesion and other biological processes. Ecm is composed of structural substances and functional macromolecular complexes, and plays a key role in the functional stabilization of cell and tissue structures^22,23^. Ecm is produced by cell secretion, In turn, it regulates cell proliferation, metastasis, and invasion. As a transmembrane glycoprotein, integrin is an important molecule of the cell–ecm network, not only regulating cell–ecm function, but also related to cell signaling. Collagen is the main component of the extracellular matrix. For components, studies have found that type IV collagen induces the migration and adhesion of melanoma and breast cancer cells through α1, β1, and α3β1 integrin, and promotes tumor metastasis^24^.

On the other hand, studies have found that gastric cancer differential genes are mainly concentrated in extracellular matrix receptor interactions, focal adhesions, PI3K-AKT and other signal pathways. Among them, focal adhesions can transmit cell adhesion signals and regulate the reorganization of the cytoskeleton. For example, tumor angiogenesis and metastasis play an important role^25^. The PI3K-AKT signaling pathway plays an important role in the regulation of cell growth, protein translation, and apoptosis^26^. A recent phase II clinical trial investigated AKT inhibition agent ipatasertib combined with paclitaxel in the treatment of triple-negative breast cancer can prolong the progression-free survival of patients^27^. Therefore, the enrichment signal pathways in this study are closely related to the metastasis and growth of cancer cells, reflecting the obvious gastric cancer tissue. The pathological changes of cancer also suggest the feasibility of targeted therapy for gastric cancer based on the above signal pathways.

## Conclusion

To sum up, this study used multiple bioinformatics methods to examine the differences in gene expression and biological processes between gastric cancer tissues and normal tissues from multiple angles. From the genetic level, we explored the malignant mechanism of gastric cancer and potential therapeutic targets. At the same time, the preliminary exploration of the regulation and interaction network of key proteins of gastric cancer provides a biological basis and data support for the precise treatment of gastric cancer.

## Data Availability

All data are available. Please contact us to access if it is needed.
